# Occurrence of mycotoxins in swine feed from South Korea

**DOI:** 10.5455/javar.2024.k756

**Published:** 2024-03-31

**Authors:** Wen Jin, Soo-Yeon Park, Yo-Han Kim, Sung-Jae Kim, Jeong-Hee Han

**Affiliations:** 1 Jiansu Agri Animal Husbandry Vocational College, Taizhou, China; 2 College of Veterinary Medicine and Institute of Veterinary Science, Kangwon National University, Chuncheon, Korea; 3 Department of Companion Animal Health, Kyungbok University, Namyangju, Korea; † These two authors contributed equally to this work.

**Keywords:** Mycotoxin, swine farm, feed, Afla, ZEN, DON, FUM, OTA

## Abstract

**Objectives:**

To update recent information on contamination levels of mycotoxins in South Korea.

**Materials and methods:**

A total of 208 samples sourced from the feeds of swine farms were collected. The contamination levels of mycotoxins, which are aflatoxin (Afla), ochratoxin A (OTA), deoxynivalenol (DON), zearalenone (ZEN), fumonisin (FUM), and T-2 toxin, were investigated by enzyme-linked immunosorbent assays (ELISAs).

**Results:**

The detection levels of the total samples were 78.91% for DON, 75.24% for Afla, 47.02% for ZEN, 68.31% for FUM, and 5.94% for OTA and T-2, which were not detected at all. Most of the analyzed mycotoxins showed significant high occurrences in 47.02%–78.91% of the swine feed samples. 11 of the 152 alfa-positive samples exceeded the maximum residue limit (MRL) of Afla proposed by the Korean regulation. In the analysis of mycotoxin detection levels by growth stage, ZEN was found in the nursery stage at a remarkably high concentration level (126.46 ± 63.76 ppb), exceeding the MRL of ZEN for piglets proposed by the European Commission. This mycotoxin was also found in the samples from the gestation barn (89.04 ± 46.05 ppb) and the farrowing house (105.58 ± 94.12) at a high concentration level. Afla was found in the nursery stage at a high concentration (8.00 ± 2.22 ppb), approaching the MRL (10 ppb) of Afla proposed by the Korean regulation.

**Conclusion:**

These results indicate that many swine farms in South Korea are still exposed to mycotoxin risk, and special attention and surveillance are necessary for these mycotoxin risks in swine farms.

## Introduction

Mycotoxins, which were toxic secondary metabolites, were produced by fungi growing on crops in the field, during handling, and in storage. The major well-known mycotoxin-producing fungi are *Aspergillus (A.) flavus, Aspergillus ochraceus***,** *Penicillium (P.) verrucosum, Fusarium (F.) graminearum,* and *Fusarium verticillioides* [1]. Cereal plants may be contaminated by mycotoxins in two ways: by fungi growing as pathogens on plants or by growing saprophytically on stored plants [2].

So far, about 300–400 mycotoxins have been known, according to the classification of compounds [3], Only a few mycotoxins have been indicated to cause significant, detrimental health and performance problems in pigs fed mycotoxin-contaminated feedstuffs. These mycotoxins that occur naturally in agricultural products are aflatoxin (Afla) produced by *A. flavus*; ochratoxin A (OTA) produced by *A. ochraceus* and *P. verrucosum*; deoxynivalenol (DON), zearalenone (ZEN), fumonisin (FUM), and T-2 toxin produced by *Fusarium spp.* [4]. Afla and OTA, as well as FUM, have been shown to cause immunosuppression, decreased feed intake, and feed refusal, a decreased feed conversion rate, and growth rates. This may lead to increased susceptibility to disease, decreased resistance of species to disease in the herd, and the possible failure of vaccination programs. Pigs consuming mycotoxins may suffer from symptoms ranging from liver and kidney failures and immunosuppression caused by Afla [5], porcine nephropathy caused by ochratoxin [6], diarrhea, vomiting, and gastrointestinal inflammation caused by DON [7], hypoestrogenism, abortion, infertility, and ulceration caused by ZEN [8], pulmonary edema syndrome, porcine pulmonary edema syndrome, hydrothorax, and thorax swelling of pigs caused by FUM [9], and oral and gastric ulcers caused by T-2 [10].

In addition, combinations of certain mycotoxins in feed ingredients and mixed feed can act synergistically to produce more pronounced detrimental effects on the animal’s performance than are normally expected for each of the mycotoxin levels evaluated individually [11]. Several mycotoxins can also exist in conjugated forms. For instance, DON and ZEN produced by Fusarium fungi, which infect crops, can exist as glucoside or glucopyranoside conjugated forms [12,13]. Moreover, a previous study indicated that bound FUM forms were more abundant than those of free FUM in European corn and corn-based foods [14].

In South Korea, most of the raw materials for animal feed depend on import, so there may be a higher risk of mycotoxin contamination. However, researchers have not thoroughly investigated the levels of mycotoxin contamination in swine farms. The present study was performed to update the latest information on mycotoxin contamination levels in swine farms through the detection of mycotoxins in swine feeds.

## Materials and Methods

### Sampling

Sampling is an important step for correct analytical results in mycotoxin determination in feed because sampling bulk feeds is difficult and mycotoxin is not evenly distributed throughout the feed to be sampled. Most errors associated with measuring mycotoxin in feed can be attributed to sampling methods. To obtain a representative sample and minimize the sampling error, the following guidelines were adopted: take multiple repeat samples from different locations in the feeders. The multiple repeat samples (1 kg per sample) were thoroughly mixed, and a subsample of 1 kg was collected from the large mixed sample at 4°C for further mycotoxin analysis [15]. The collection of samples was carried out for 2 months, from August 2021 to October 2021.

### Sample preparation

**Figure 1.**
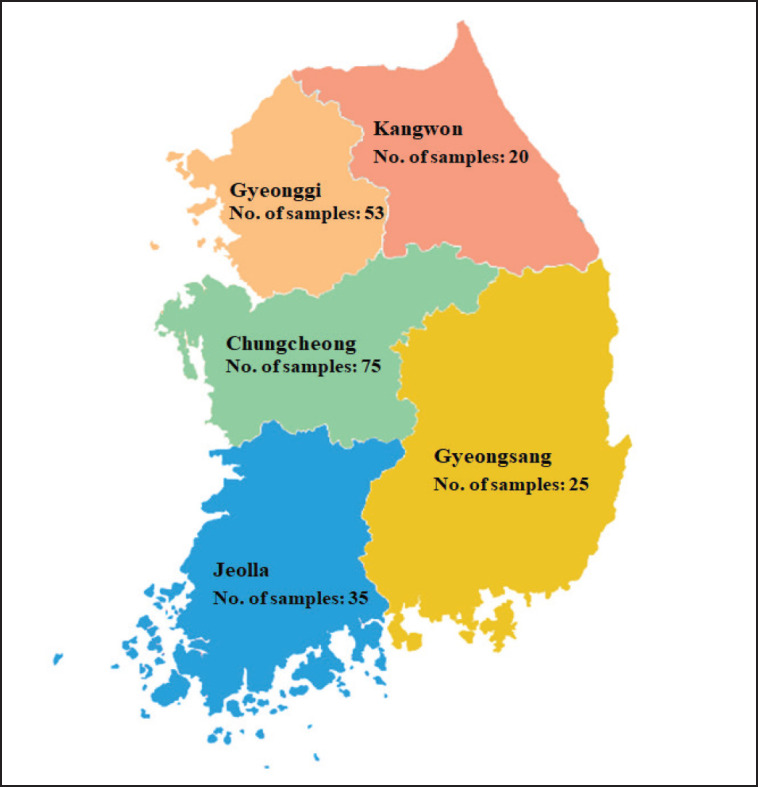
The distribution of sampling by five regions of South Korea in this study. A total of 202 samples includes 20 of Kangwon, 53 of Gyeonggi, 75 of Chungcheong, 35 of Jeolla, and 25 of Gyeongsang province.

A total of 202 feed analytical samples were collected from 45 swine farms distributed in five regions of South Korea (Kangwon, Gyeonggi, Chungcheong, Jeolla, and Gyeongsang provinces). Four to five samples per farm originating from different stages of pig production, such as nursery, finish, fattening room, gestation barn, and farrowing house, were taken directly at different places. Based on geographical origin, 202 samples received consisted of 20 from Kangwon, 52 from Gyeonggi, 70 from Chungcheong, 35 from Jeolla, and 25 from Gyeongsang Province. Based on the growth stages of pig production, all were composed of 43 nurseries, 43 finishing rooms, 31 fattening rooms, 42 gestation barns, and 43 farrowing house samples. By feeder type, only 71 of the 202 samples were confirmed, including 41 dry feeders and 30 wet/dry feeders (Fig. 1).

### Detection of mycotoxin

All samples were analyzed by Quantas Analytics, a subsidiary company of Romer Labs Diagnostic GmbH, which specializes in feed analysis. Afla, OTA, DON, ZEN, FUM, and T-2 extractions were performed according to the manufacturer’s instructions for enzyme-linked immunosorbent assays (ELISA). Twenty grams of sample were extracted with 100 ml of 70% methanol for Afla, ochratoxin, ZEN, and T-2 and with 100 ml of distilled water for DON. The extracts were filtered, and 1 ml of filtrate was diluted with 1 ml of distilled water except for DON, for which no dilution was made. The extract was filtered through a Whatman #1 filter paper, and the filtered sample was then diluted with distilled water. About 100 μl of diluted filtrate per well was used for the ELISA test. The optical density was measured at a wavelength of 450 nm using an ELISA microplate reader 680 (USA, Bio-Rad) with RIDASCREEN software for evaluation of ELISA data and the mycotoxin concentrations. To get a better overview of mycotoxin occurrence in South Korea, data were analyzed as follows: geographical regions, means of growth stages of pig production, and feeder types.

## Results

### Overall contamination levels of mycotoxins in feeds

The MRLs of mycotoxins proposed by the Korean regulation for feedstuffs and the European regulations EC No. 32/2002 and EC No. 576/2007 (Korea Food and Drug Administration, 2007; The European Commission, 2002 and 2007) are shown in Table 1. As shown in Table 2, from a total of 202 samples from 45 farms, Afla, OTA, ZEN, FUM, and DON were found in 75.24% (152), 5.94% (12), 47.02% (95), 68.31% (138), and 78.71% (159) with concentrations of 7.67 ± 1.86, 5.10 ± 2.87, 128.32 ± 99.04, 880.00 ± 800, and 510.00 ± 210 ppb, respectively. T-2 was not detected in all tested samples. 11 of the 152 alfa-positive samples exceeded the maximum residue limits (MRL) (10 ppb) of Afla proposed by the Korea regulation. From 6 of these 11 samples, ZEN, FUM, and DON were also detected together. The concentration of OTA was 5.10 ± 2.87 ppb, far below the MRL (200 ppb) for OTA of the Korean regulation.

### Contamination levels of mycotoxins by geographic region

The contamination levels of the samples from Kangwon province were relatively severe compared to other regions (Gyeonggi, Chungcheong, Gyeongasng, and Jeolla), although OTA was not detected at all. For Afla, ZEN, FUM, and DON, 95, 80, 100, and 95 of the samples were positive with 8.38 ± 1.40, 160.88 ± 98.03, 620.00 ± 281.18, and 580.00 ± 195.25 ppb, respectively (Table 3). Regionally, Kangwon province exhibited the highest detection rates of mycotoxins detected in this study, except for OTA.

Whereas Jeolla province exhibited the best overall condition among all regions. For Afla, OTA, ZEN, FUM, and DON, 58.82%, 5.88%, 29.41%, 44.11%, and 61.76% of samples were positive with 7.71 ± 1.66, 2.36 ± 0.06, 74.03 ± 33.22, 650.00 ± 420.10, and 380.00 ± 135.53 ppb, respectively (Table 4). The detection rates of mycotoxins were the lowest across the country, except for FUM and OTA. In the case of FUM, Jeolla province showed the second lowest detection rates at 44%, similar to 40% in Gyeongsang province. OTA exhibited very low levels of detection rate and concentration compared to other mycotoxins, so it was considered a negligible factor in regional comparisons.

### Contamination levels of mycotoxins by growth stage

Among the detected mycotoxins, DON exhibited relatively high detection rates across all stages. The detection rates at the stages of nursery, finish, fattening, gestation barn, and farrowing house were 81.39%, 82.66%, 80.00%, 80.95%, and 76.74%, respectively.

**Table 1.** MRLs proposed for mycotoxin in animal feeds from Korea and European commission.

Country	Mycotoxin	Maximum residue limit in μg/kg (ppb)	Products intended for animal feed
Korea	Afla (B1, B2, G1, G2)	10	Complete feedstuffs for pig, calves, dairy cattle, chicken
	OTA	200	Complete feedstuffs except for premix feedstuffs
		200	Simple feedstuffs
European Commission	Afla B1	50	Complete feeding stuffs for pig, cattle, sheep and goats except for the young animals
	Deoxynivalenol	900	Complementary and complete feeding stuffs for pigs
	ZEN	100	Complementary and complete feeding stuffs for piglets and gilts (young sows)
		250	Complementary and complete feeding stuffs for fattening pigs and sows
	OTA	50	Complementary and complete feeding stuffs for pigs
	FUM B1 + B2	5,000	Complementary and complete feeding stuffs for pigs

**Table 2.** Overall levels of mycotoxin contamination in feeds.

Mycotoxin	No. of detected	Detection rate (%)	Mean ± SD (ppb)	Contamination values range (μg/kg)
Afla	152	75.24	7.67 ± 1.86	0–12.09
OTA	12	5.94	5.10 ± 2.87	0–11.54
ZEN	95	47.02	128.32 ± 99.04	0–719.42
FUM	138	68.31	880.00 ± 800	0–7,910.00
T-2	0	0	ND	ND
DON	159	78.71	510.00 ± 210	0–1,310.00

**Table 3.** Mycotoxin concentrations and detection rates by geographic region.

Mycotoxin	Province^*^	Mean ± SD (ppb)	Detection rates (%)
Kangwon	Afla	8.38 ± 1.40	95.00
	OTA	0	0
	ZEN	160.88 ± 98.03	80
	FUM	620.00 ± 281.18	100
	DON	580.00 ± 195.25	95.00
Gyeonggi	Afla	7.07 ± 2.22	62.26
	OTA	4.85 ± 2.29	11.32
	ZEN	73.86 ± 33.79	41.50
	FUM	920.00 ± 589.33	66.03
	DON	500.00 ± 174.85	83.01
Chungcheong	Afla	7.68 ± 1.67	78.66
	OTA	9.49 ± 2.89	2.66
	ZEN	109.50 ± 89.87	44.00
	FUM	990.00 ± 565.54	76
	DON	540.00 ± 243.28	82.66
Gyeongsang	Afla	8.15 ± 1.95	92
	OTA	4.20 ± 0.56	8
	ZEN	122.74 ± 154.95	72
	FUM	720.00 ± 304.09	40
	DON	490.00 ± 129.19	68
Jeolla	Afla	7.71 ± 1.66	58.82
	OTA	2.36 ± 0.06	5.88
	ZEN	74.03 ± 33.22	29.41
	FUM	650.00 ± 420.10	44.11
	DON	380.00 ± 135.53	61.76

**Table 4.** Mycotoxin detection rates according to growth stages.

Growth stages	Mycotoxin	Mean ± SD (ppb)	Detection rates (%)
Nursery	Afla	8.00 ± 2.22	62.79
	OTA	4.98 ± 1.13	6.97
	ZEN	126.46 ± 63.76	37.20
	FUM	760.00 ± 367.20	65.11
	DON	520.00 ± 155.27	81.39
Fattening	Afla	7.92 ± 1.99	80.00
	OTA	6.58 ± 3.76	13.33
	ZEN	74.94 ± 34.58	46.66
	FUM	860.00 ± 596.49	63.33
	DON	540.00 ± 243.28	82.66
Finish	Afla	7.32 ± 2.03	72.09
	OTA	3.32 ± 1.82	4.65
	ZEN	222.00 ± 160.93	51.16
	FUM	1020.00 ± 442.54	69.76
	DON	570.00 ± 312.41	80.00
Gestation barn	Afla	7.75 ± 1.47	90.47
	OTA	4.44 ± 2.60	7.14
	ZEN	89.04 ± 46.05	42.85
	FUM	690.00 ± 416.37	66.66
	DON	490.00 ± 162.03	80.95
Farrowing house	Afla	7.45 ± 1.67	74.41
	OTA	0	0
	ZEN	105.58 ± 94.12	58.13
	FUM	960.00 ± 719.86	74.41
	DON	520.00 ± 218.28	76.74

In the results of the ZEN analysis, 37.2% of the samples that originated from the nursery stage were positive with a high concentration (126.46 ± 63.76 ppb). This level exceeded the MRL (100 ppb) of ZEN for piglets proposed by the European Commission. Also, ZEN exhibited a high concentration (222.00 ± 160.93 ppb) approaching the MRL for fattening pigs (250 ppb), proposed by the European Commission, in the samples originated from the finish stage with 51.16% of the detection rate (Table 4).

In the results of the Afla analysis, the detection rates at the stages of nursery, finish, fattening, gestation barn, and farrowing house were 62.8%, 72.1%, 80%, 90.5%, and 74.4%, respectively, and the detection rate of gestation barn was the highest among the stages. Afla exhibited relatively high concentrations across all stages. The concentrations at the stages of nursery, finish, fattening, gestation barn, and farrowing house were 8.00 ± 2.22, 7.92 ± 1.99, 7.32 ± 2.03, 7.75 ± 1.47, and 7.45 ± 1.67 ppb. Especially, the concentration of the nursery stage approached the level of 80% of the MRL (10 ppb) of Afla proposed by the Korean regulation.

### Contamination levels of mycotoxins by feed type

The detection rates of Afla, OTA, FUM, and DON in wet/dry feeders were 83.33%, 13.33%, 86.66%, and 76.66%, with 7.72 ± 1.80, 7.90 ± 4.04, 890 ± 605.27, and 610 ± 283.00 ppb. Most of the detected mycotoxins except for ZEN had relatively higher prevalence and concentrations in the wet/dry feeder compared to the dry feeder (Table 5).

## Discussion

Fungi are ubiquitous, and various mycotoxins from fungi can contaminate all feedstuffs in all segments of the animal feed supply chain. The major problem of mycotoxin contamination in animal feed is usually not acute disease episodes but low-level toxin ingestion, which can cause metabolic disturbances resulting in poor productivity [16]. Rodrigues and Naehrer (2012) reported that Afla, ZEN, DON, FUM, and OTA were found in 33%, 45%, 59%, 64%, and 28% of analyzed worldwide samples (feedstuffs and feed) between 2009 and 2011 [17]. In this study, Afla, ZEN, DON, FUM, and OTA were found in 75%, 47%, 78%, 68%, and 6% of the analyzed samples, and most of the mycotoxins except for OTA presented more severe contamination levels even compared to the worldwide results from 10 years ago. Moreover, 11 of the 152 alfa-positive samples exceeded the MRL (10 ppb) of Afla proposed by the Korean regulation, and 6 of these 11 samples presented complex contamination of Afla, ZEN, FUM, and DON. These results indicate that many swine farms in South Korea are still exposed to mycotoxin risk.

**Table 5.** Mycotoxin detection rates according to feeder type.

Feeder type	Mycotoxins	Mean(ppb) ± SD	Detection rates (%)
Wet/dry	Afla	7.72 ± 1.80	83.33
	OTA	7.90 ± 4.04	13.33
	ZEN	107.30 ± 83.52	36.66
	FUM	890.00 ± 605.27	86.66
	DON	610.00 ± 283.00	76.66
Dry	Afla	7.57 ± 1.76	70.73
	OTA	2.36 ± 0.06	4.87
	ZEN	164.90 ± 79.64	43.90
	FUM	670.00 ± 350.78	75.60
	DON	540.00 ± 176.92	63.41

DON has great economic importance in worldwide feed grain use because it is well documented as a cause of feed refusal or reduced feed intake in swine [18,19]. In this study, a total of 202 samples were analyzed, and the occurrence of DON was the highest, with 78.91% positive. This result was consistent with DON was highly prevalent in North Asia [20]. Other detected mycotoxins except OTA also showed high occurrences (47.02%–78.71% of the total feed samples).

When looking at the contamination levels of mycotoxins by growth stage, except for OTA, most of the detected mycotoxins (Alfa, ZEN, FUM, and DON) showed high occurrences of 37.20%–82.66%. These results indicate that pigs of all stages are exposed to a variety of mycotoxin risks.

Specifically, ZEN was found in the nursery stage at a remarkably high concentration level (126.46 ± 63.76 ppb), exceeding the MRL (100 ppb) of ZEN for piglets proposed by the European Commission, although the detection rate was relatively low at 33.2%. This mycotoxin was also found in the samples from the gestation barn (89.04 ± 46.05 ppb) and the farrowing house (105.58 ± 94.12) at a somewhat high concentration level, with occurrences of 42.85% and 58.13%, respectively. ZEN, which can play an estrogen analog, binds competitively to estrogen receptors of the uterus, mammary gland, liver, and hypothalamus and, as a result, hampers reproductivity in pigs [21]. Such high contamination of ZEN in the feed from the nursery stage, gestation barn, and farrowing house can cause health problems for sucking and post-weaning piglets with hyperestrogenism and reduced reproductivity in sows [22].

Afla, which is a strong hepatotoxic substance [23], showed the second highest occurrence (75.24%), at a similar level to DON (78.71%). Especially, among the mycotoxin detection rates by growth stage, the occurrence of Alfa in the gestation barn, where sows stay during the pregnancy period, was remarkably high at 90.47%. Also, this toxin was found in the nursery stage at a high concentration (8.00 ± 2.22 ppb), approaching the MRL (10 ppb) of Afla proposed by the Korean regulation, with a somewhat high occurrence of 62.79%. Piglets and pregnant pigs are the most susceptible to mycotoxins [24,25]. The contamination of ZEN and Afla in the feeds from the nursery stage, gestation barn, and farrowing house appears to be at risk level considering both detection rate and concentration, and it is likely to cause significant damage in swine farms. Thus, special attention and surveillance are necessary for these mycotoxins on swine farms.

Moisture and temperature are major factors in fungus growth and mycotoxin production. High temperatures and moisture levels promote mycotoxin growth during storage and transportation [26]. In this study, the samples from the wet/dry feeder presented relatively high mycotoxin contamination levels compared to the samples from the dry type, which was likely attributed to humidity differences between the two feeder types.

However, the regional analysis of mycotoxin contamination showed results contrary to those above. The highest contamination levels of Afla, ZEN, FUM, and DON were all found in Kangwon province, and it was estimated that Kangwon province was the most prevalent mycotoxin among other regions. Kangwon Province is a relatively cold and dry region compared to other regions in South Korea. Rather, Jeolla-do, a humid and warm region [27], showed the lowest contamination level among the regions. Thus, regional climate differences do not appear to have contributed significantly to mycotoxin contamination. Raising pigs in Kangwon province takes up 3%–4% of the whole country. More than half of those farms are small-scale, with less than 100 sows, and such small-scale farms are likely to have poor management systems and facilities. It is assumed that regional differences in mycotoxin contamination in South Korea are attributed to inadequate feed management rather than a climate factor.

## Conclusion

In this study, the contamination levels of problematic mycotoxins (Afla, OTA, DON, ZEN, FUM, and T-2) were investigated in the feeds collected from swine farms in South Korea. All tested mycotoxins except T-2 were detected in the analyzed feed samples, and most of the mycotoxins showed high contamination levels. These results indicate that many swine farms in South Korea are still exposed to mycotoxin risk, and special attention and surveillance for mycotoxin in swine feeds are necessary.

## List of Abbreviations

Afla, aflatoxin; OTA, ochratoxin A; ZEN, zearalenone; FUM, fumonisin; DON, deoxynivalenol; ELISA, enzyme-linked immunosorbent assay; MRL, maximum residue limit.
